# Enhancing the Selection and Performance of Working Dogs

**DOI:** 10.3389/fvets.2021.644431

**Published:** 2021-05-12

**Authors:** Emily E. Bray, Cynthia M. Otto, Monique A. R. Udell, Nathaniel J. Hall, Angie M. Johnston, Evan L. MacLean

**Affiliations:** ^1^Arizona Canine Cognition Center, School of Anthropology, University of Arizona, Tucson, AZ, United States; ^2^Canine Companions for Independence, National Headquarters, Santa Rosa, CA, United States; ^3^Penn Vet Working Dog Center, Department of Clinical Sciences and Advanced Medicine, School of Veterinary Medicine, University of Pennsylvania, Philadelphia, PA, United States; ^4^Human-Animal Interaction Laboratory, Department of Animal and Rangeland Sciences, Oregon State University, Corvallis, OR, United States; ^5^Canine Olfaction Lab, Department of Animal and Food Science, Texas Tech University, Lubbock, TX, United States; ^6^Boston College Canine Cognition Center, Psychology and Neuroscience Department, Boston College, Chestnut Hill, MA, United States; ^7^Cognitive Science Program, University of Arizona, Tucson, AZ, United States; ^8^Department of Psychology, University of Arizona, Tucson, AZ, United States; ^9^College of Veterinary Medicine, University of Arizona, Tucson, AZ, United States

**Keywords:** assistance dogs, canine, detection dogs, selection, temperament, working dogs, protection dogs

## Abstract

Dogs perform a variety of integral roles in our society, engaging in work ranging from assistance (e.g., service dogs, guide dogs) and therapy to detection (e.g., search-and-rescue dogs, explosive detection dogs) and protection (e.g., military and law enforcement dogs). However, success in these roles, which requires dogs to meet challenging behavioral criteria and to undergo extensive training, is far from guaranteed. Therefore, enhancing the selection process is critical for the effectiveness and efficiency of working dog programs and has the potential to optimize how resources are invested in these programs, increase the number of available working dogs, and improve working dog welfare. In this paper, we review two main approaches for achieving this goal: (1) developing selection tests and criteria that can efficiently and effectively identify ideal candidates from the overall pool of candidate dogs, and (2) developing approaches to enhance performance, both at the individual and population level, via improvements in rearing, training, and breeding. We summarize key findings from the empirical literature regarding best practices for assessing, selecting, and improving working dogs, and conclude with future steps and recommendations for working dog organizations, breeders, trainers, and researchers.

Since their domestication more than 10,000 years ago (1–3), the nature of dogs' interactions with people has taken many forms. On one end of the spectrum, free-ranging dogs live largely on the outskirts of society, interacting minimally with humans other than to scavenge for food (4). On the other end of the spectrum, pet dogs are welcomed into our homes (5) and beds (6), valued for their companionship, and can evoke emotional reactions analogous to those in the parent-child bond (7–9). Within this patchwork of human-dog interconnectedness, working dogs represent a small subset of the dog population, but one that can have profound effects on human health and well-being.

The roles that working dogs perform, now and throughout history, have been extremely diverse. Dogs have played critical roles in hunting and agriculture [e.g., livestock guarding dogs, herding dogs; (10)], transportation [e.g., sled dogs; (11)], public health [e.g., medical detection dogs; (12)], and environmental protection [e.g., conservation dogs; (13, 14)]. Although dogs working in each of these areas provide important benefits to humans, a comprehensive review of the many roles that working dogs fulfill is beyond the scope of the current paper. Thus, in this review, we focus on working dogs employed in three of the most common applications: assistance, protection, and detection dogs.

The primary purpose of an assistance dog is to perform tasks for an individual with a disability that ultimately allows that person to achieve greater independence. Research suggests that these dogs are effective not only as mobility aids, but may also provide psychosocial benefits to their partners (15–25). Assistance dogs represent a more recently-developed working dog role (26). While the first assistance dog organization was established in the United States with the opening of The Seeing Eye guide dog school in 1929, assistance dog use became more widespread only as recently as 1990 (27), when the Americans with Disabilities Act protected the right of a service dog to accompany their partner to public places (28). As of 2020, there are 133 assistance dog providers accredited by Assistance Dogs International, Inc.^1^ There are several distinct categories of placements that fall under the umbrella of “assistance dogs”: guide dogs, who assist blind or visually impaired handlers with navigation of their environment; hearing dogs, who assist people who are deaf or hard of hearing by alerting to relevant sounds (27), and service dogs, who assist people with physical disabilities by helping with daily tasks such as opening doors and retrieving objects. In more recent years, service dogs have also been trained to assist people with Autism Spectrum Disorder (ASD) under the supervision of a third party, usually a parent (29), to assist Veterans suffering from posttraumatic stress disorder (30), and to use olfaction to alert their handler to relevant medical events (31).

In contrast to assistance dogs, the title therapy dog is often given to dogs trained to help people in other settings ranging from facilitating children's practice reading (32), to promoting social interactions among the elderly (33), or participating in psychosocial interventions for children with disabilities (34). While therapy dogs often provide directed services for a human handler, they do not have legally protected access to accompany their owner/handler into businesses and public places (35). Some therapy dogs are further distinguished from assistance dogs, as described above, in that their handler may not be the direct beneficiary of the dog's presence, but rather a facilitator of the dog's interactions with others, often in a health care or school setting. These therapy dogs may participate in animal-assisted interventions, a broad category of tasks which can refer to either animal-assisted therapy—undertaken in conjunction with a health professional, working toward a specific goal—or animal-assisted activity—undertaken in conjunction with a professional or volunteer in a more spontaneous setting (36). Although therapy dogs play important roles, this topic has been covered in depth elsewhere [e.g., (37, 38)], and so the current paper focuses solely on assistance, protection, and detection dogs.

Reports of using dogs for protection can be found as early as 700 BC, but the advent of modern police dogs only dates back to the early 20th century (39). Police dogs are used in law enforcement to aid in the apprehension of suspects, deterrence of crime, securing of points of entry, and locating of people or substances of interest. Similarly, the military trains single-purpose patrol dogs to scout, search buildings, and use controlled aggression. In addition, single-purpose detection dogs serve to locate explosives, narcotics, contraband, pests, and many more odors [e.g., (40, 41)]. Many law enforcement agencies and the military also rely on dual-purpose dogs who are used for both protection and detection. Search-and-rescue dogs are trained to find either live humans or human remains and can be trained for response in urban disaster settings or wide-area wilderness settings (42).

Thus, from helping people with disabilities safely and confidently navigate their environment to aiding in the safeguarding of our communities, working dogs provide numerous benefits at both the individual and societal level. They not only fulfill these key needs—often outperforming technologies designed for the same purposes—but they also enhance the lives of the people with whom they work through the human-animal bond (18, 43, 44). However, the process of identifying and training dogs with potential for success in these roles presents many challenges.

## Sourcing of Dogs

The first hurdle is determining how to source the dogs. There are several common models, all of which have their own advantages and disadvantages. Many assistance dog organizations (45) as well as some military dog organizations [e.g., the Swedish Armed Forces; (46)] maintain their own breeding programs, which is beneficial for several reasons. Breeding decisions can be informed by generations of information, affording organizations greater control over the health and characteristics of their dogs. For example, Guiding Eyes for the Blind reports that, through selective breeding, they have decreased the rates of hip and elbow dysplasia in their population by over 90% in the past 30 years (47). Also, organizations with their own breeding programs report the highest success rates for dogs from their own breeding programs (48, 49). However, dogs still need to occasionally be brought in from outside sources to maintain genetic diversity. Furthermore, breeding programs can be costly. One approach to improve success in breeding programs is to adopt a cooperative approach in which dogs who are suited for other careers are exchanged with complementary organizations (50).

The traditional approach to sourcing dogs for search and rescue is a community-based model (51). In this setting, individuals identify a dog with the potential physical and behavioral characteristics appropriate for a search dog (52). This individual approach may include identifying breeders with a history of success or simply a trial-and-error approach with individual dogs. This approach is labor intensive for the individual and results in a variable success rate. Recently, some organizations have established programs in which trained search dogs from select breedings are available for pairing with handlers (e.g., Penn Vet Working Dog Center, Maranatha Kennels).

Another option which has great public appeal is to identify dogs from shelters or rescues to be trained as working dogs (51). This approach serves the double purpose of fulfilling a working need and providing a home to a dog. In theory, it represents a lower cost model, given that organizations are not responsible for the breeding and rearing of candidate dogs. However, this model has several challenges with identifying and training dogs to become successful working dogs. While medical conditions are (usually) readily screened, behavioral potential is difficult to evaluate (53, 54). Furthermore, dogs are often placed in shelters or rescues due to behavioral problems (e.g., fear associated with the environment or people, resource guarding, dog- or human-directed aggression) that are unsuitable for working dogs (55, 56). Although some programs have been successful in this approach, the financial investment can be greater than that for a breeding program or other acquisition approaches, especially if the organization maintains responsibility for adopted dogs who do not meet the working dog requirements. Thus, utilization of shelter and rescue dogs is best seen as a complementary approach until improved screening can be developed to clearly identify candidates that possess the physical and behavioral traits to be successful in a specific working career.

Finally, many smaller assistance dog organizations purchase dogs (57), and it is also common for the military to procure dogs from overseas (58). When acquiring dogs, the organization has the advantage of selecting only dogs who meet the physical and behavioral requirements, but current behavioral tests are imperfect and still result in a sizable proportion of dogs being subsequently rejected for behavioral reasons (57).

## Attrition From Training

An additional obstacle is that, even after undergoing rigorous selection and training, large numbers of dogs who enter training fail to complete these programs, largely for behavioral reasons (59–61). The consequences of unsuccessful dogs are numerous. Often, dogs are not deemed unsuitable until a year or two of age, at which point large amounts of time and money have already been invested in them. It is estimated that around 50–70% of assistance dogs are ultimately released from professional training programs (62), and the release rate can be as high as 80% for dogs acquired from a shelter (63). In addition to improvements in resource allocation, increasing the success rate of dogs in training also has welfare implications (64). For example, dogs bred for placement in protection roles often have characteristics, such as high motivation (sometimes referred to as “drive”), reactivity, and energy levels, that make them difficult to keep as pets. Similarly, accurate identification of dogs who are unlikely to succeed in working roles can eliminate potentially stressful transitions that these dogs would otherwise face (e.g., beginning a professional training program only to be rehomed shortly after initiation of this process).

To address these challenges, we advocate multiple avenues by which to improve the process of producing effective and healthy working dogs, that can ultimately lead to the placement of more dogs with a greater potential for success and welfare in these roles. We summarize key findings from the empirical literature regarding best practices for assessing, selecting, and improving working dogs, and conclude with future steps and recommendations for working dog organizations, breeders, trainers, and researchers.

## What Factors Will Optimize the Process of Placing Successful Working Dogs?

### Assessment and Selection of Working Dogs

One opportunity to optimize the production of working dogs occurs at the stage of deciding which dogs to train for working roles. The goal here is to refine and improve predictions of which dogs will ultimately complete training, and beyond that, thrive throughout long and productive careers. In practice, these evaluations can act as tools for information-gathering across relevant domains, but how they are applied and the subsequent cost-benefit analyses will vary based on the specific industry, the size of a given organization, the age at which training starts, and the origin of the dogs (e.g., from a breeding colony, private breeder, shelter). Importantly, implementation of these tests and criteria are meant to identify suitable candidates and thereby make selection more efficient, but would not result in improvement to the overall pool of dogs from which future candidates could be selected. Ideally, the selection process would take into account multiple factors that affect a dog's working ability, including various facets of the dog's behavior and cognition, early environment, and preferences. Below, we highlight factors linked to success, as indicated by prior research, in each of these areas.

#### Behavioral Considerations

Behavior is a major factor when it comes to placing working dogs. Accordingly, there has been a large research focus on determining which specific elements of canine behavior are necessary, and which are disruptive, when it comes to the ability of working dogs to perform their roles. Several reviews have been written about this topic in recent years, including an assessment of the behavioral tests used to select assistance, protection, and detection dogs (65), as well as other papers summarizing the behavioral and cognitive features believed to be important in the selection of detection dogs specifically (66–68). Additionally, studies have documented the qualities deemed most important for detection dogs by their handlers and trainers (69, 70).

In Table 1, we summarize 33 empirical studies that have assessed behavior in candidate (ages 3 months to 4 years) and active (ages 2.5–11 years) working dogs in order to determine associations with a work-related outcome. In addition to focusing on participants of a specified age range (i.e., 3 months and older) and with a reported outcome associated with a specified role (i.e., assistance, detection, and protection), included studies were published between January 1983 and November 2020. In the vast majority of these studies, the outcome metric used was qualification—i.e., successful placement—as an assistance, protection, and/or detection dog, with the alternative being that the dog was deemed unsuitable and released for behavioral shortcomings. For a handful of studies, the outcome measures were instead performance on specific job-related tests [e.g., on working dog trials: (94); on a Military Working Dog suitability test: (95); on an annual search test: (93)] or trainer assessment of ability (63, 82, 85, 96). And finally, two studies addressed the longer-term efficacy of working dogs and therefore only included subjects that were already initially placed as detection dogs; the first study investigated factors contributing to the longevity of a successful working dog career by comparing active dogs vs. those that had qualified but were subsequently withdrawn from service (61), while the other analyzed what behavioral features predicted speed of drug detection (92). Although not every aspect of behavior measured in each of these studies predicted success, at least a subset of measures in all of these studies had some predictive validity.

**Table 1:** Associations between behavior and outcomes in adolescent and adult candidate working dogs.

**Paper**	**Authors**	**Year**	**Reference**	**Outcome**	**Breeds**	**Age at assessment**	***n***	**Measurement type**	**Behavioral traits related to outcome (direction of association with success)**
a	Goddard and Beilharz	1983	(49)	Assistance: guide dog qualification	LR and GR	12–18 months	887	Ratings by trainers (of behavior over 3 weeks)	Fearfulness (−), distraction (−), aggression (−)
b	Wilsson and Sundgren	1997	(71)	Assistance: guide dog qualification	LR and GSD	450–600 days	2,107	Behavioral assessment	Ability to cooperate (+), courage (+, GSD only), nerve stability (+, GSD only)
c	Batt et al.	2008	(72)	Assistance: guide dog qualification	LR and GR	6 and 14–20 months	43	Behavioral assessment	Shorter latency to drop during passive test (+), greater latency to rest during passive test (+), absence of jumping during dog distraction task (+), higher lateralization index during tape test (+), lower rate of both paw usage during Kong test (+), lack of pulling during dog distraction task (+)
d	Arata et al.	2010	(73)	Assistance: guide dog qualification	LR	15 months	144	Ratings by trainers (of behavior over 3 months)	Distraction (−), docility (+)
e	Tomkins et al.	2011	(74)	Assistance: guide dog qualification	LR, GR, and LGX	13–17 months	113	Behavioral assessment	Panting and licking during dog distraction test (−), latency to sit in noise test (−), time resting in evening kennel (+)
f	Tomkins et al.	2012	(75)	Assistance: guide dog qualification	LR, GR, and LGX	13–17 months	114	Behavioral assessment	Right-directional paw preference in Kong test (+), strength of laterality bias in first-stepping test (+)
g	Harvey et al.	2016	(76)	Assistance: guide dog qualification	LR and LGX	5 and 8 months	93	Behavioral assessment	Time oriented toward food (−), shaking behavior after body sensitivity tests (+), lip licking (−), obedience in command-following (+), reactivity (−), distraction (−), Fear/anxiety (−)
h	Harvey et al.	2017	(77)	Assistance: guide dog qualification	LR, GR, LGX, and GSD	5, 8, and 12 months	1,401	Ratings by training supervisors (of behavior over months)	Trainability (+), distractibility (−), general anxiety (−), adaptability (+), excitability (−), stair anxiety (−), body sensitivity (−)
i	Bray et al.	2017	(78)	Assistance: guide dog qualification	LR, GR, LGX, and GSD	14–17 months	98	Behavioral assessment	Problem-solving performance (+), quicker to vocalize during a novel object task (−)
j	Cleghern et al.	2018	(79)	Assistance: guide dog qualification	LR	12 and 16 months	1,561	Ratings by puppy raisers (of behavior over months, at 12 months) and behavioral assessment (at 16 months)	Aggression toward unfamiliar people (−), fearful behavior (−), nervous on stairs (−), dog aggression (−), kennel anxiety (−)
k	Duffy and Serpell	2012	(62)	Assistance: guide and service dog qualification	LR, GR, LGX, and GSD	6 and 12 months	7,696	Ratings by puppy raisers (of behavior over months)	27/36 CBARQ traits, including pulling on the leash (−), energy level (−), hyperactivity (−), fear (−), and chasing (−)
l	Dollion et al.	2019	(80)	Assistance: guide and service dog qualification	LR, BMD, LBX, SP, RP, GR, and LGX	6 and 12 months	5,340	Ratings by foster families (of behavior over months, at 6 + 12 months) and behavioral assessment (at 12 months)	Fear/reactivity (−)
m	Berns et al.	2017	(81)	Assistance: service dog qualification	LR, GR, and LGX	17–21 months	49	Awake fMRI	Caudate activity (+) and amygdala activity (−) while watching hand signals
n	MacLean and Hare	2018	(82)	Assistance: service dog qualification	LR, GR, and LGX	2 years	232	Behavioral assessment	Human-directed gazing during unsolvable and social referencing tasks (+), inferential reasoning (+)
o	Bray et al.	2019	(83)	Assistance: service dog qualification	LR, GR, and LGX	12 months	3,569	Ratings by puppy raisers (of behavior over months)	Barking (−), stranger-directed fear (−), dog-directed aggression (−), coprophagia (+), trainability (+)
o	Bray et al.	2019	(83)	Assistance: service dog qualification	LR, GR, and LGX	18 months	5,967	Behavioral assessment	Body tension during physical exam (−), reactivity during noise test (−), uncomfortable around unfamiliar dog stimulus (−), reactivity during prey test (−)
p	Weiss	2002	(63)	Assistance: trainer rating on “service success” scale	Varied	6 months−2 years	40	Behavioral assessment	High levels of vertical activity level when alone in an empty room for 4 min (−), trying to solicit interaction with a silent staring human (+)
q	Maejima et al.	2007	(84)	Detection: drug detection dog qualification	LR	1–2 years	197	Behavioral assessment	Desire for work (+): concentration, interest in target, obedience training, general activity, anxiety
r	Rooney et al.	2007	(85)	Detection: explosive detection dog trainer assessment of overall ability	LR	14–15 months	26	Behavioral assessment	Subjective measure of general search ability (+), free search thoroughness (+), location ability (+), systematic search behavior (+)
s	McGarrity et al.	2016	(86)	Detection: TSA odor-detection dog qualification	LR, V, and crosses	3, 6, 9, and 12 months	52	Behavioral assessment	Environmental stability: responsiveness (+), initiative (+), confidence (+), concentration (+); dominant possession (+); increase in hunt drive over 1st year of life (+)
t	Hare et al.	2018	(87)	Detection: search-and-rescue dog FEMA-certification	GSD, LR, GR, and assorted other breeds	1–11 years	129	Ratings by handlers (of behavior over months)	Fear of dogs (−), separation-related problems (−)
n	MacLean and Hare	2018	(82)	Detection: detection dog success	LR	4 years	312	Behavioral assessment	Sensitivity to human gesture cues (+), short-term memory (+)
u	Lazarowski et al.	2018	(88)	Detection: vapor wake® detection dog and explosive detection dog placement	LR and GWPX	3, 6, 10, and 12 months	146	Behavioral assessment	Performance (+): hunt, focus, possession, independence, work effort; environmental soundness (+): comfortable around surfaces, people, vehicles, quick recovery to visual startle, quick recovery to acoustic startle; trainability (+)
v	Lazarowski et al.	2019	(89)	Detection: detection dog qualification	LR and GWPX	3, 6, and 11 months	77	Behavioral assessment	Follow olfactory vs. deceptive social cues (+)
w	Lazarowski et al.	2019	(90)	Detection: detection dog qualification	LR and GWPX	3, 6, and 11 months	81	Behavioral assessment	Human-directed gazing during an unsolvable task (+)
x	Lazarowski et al.	2020	(91)	Detection: detection dog qualification and trainer evaluation of performance measures	LR and GWPX	3, 6, and 11 months	113	Behavioral assessment	Longer latencies to detour during first reversal trial of detour task at 3 months (+), more correct choices in acquisition phase of detour task at 11 months (+), short-term memory at 3 months (+)
y	Ganitskaya et al.	2020	(92)	Detection: speed of drug detection	LR, GR, ECS, RS, and GS	2.5–7.5 years	74	Behavioral assessment	Play (+), sociability (+), activity (+)
z	Tiira et al.	2020	(93)	Detection: police explosive search dogs annual search test success	BM, GSD, and LR	12–112 months	23	Behavioral assessment	Motor inhibition measured via cylinder task performance (+)
A	Svartberg	2002	(94)	Protection/detection: working dog trial performance	GSD and BT	12–18 months	2,655	Behavioral assessment	Boldness (+): playfulness, curiosity/fearlessness, chase-proneness, and sociability
B	Sinn et al.	2010	(58)	Protection/detection: military working dog dual-certification	GSD, BM, and DS	1–3 years	1,000	Behavioral assessment	Search focus (+), sharpness (+)
C	Wilsson and Sinn	2012	(46)	Protection/detection: Swedish Armed Forces training program success	GSD	15–18 months	496	Behavioral assessment	Engagement (+), confidence (+)
D	Foyer et al.	2014	(95)	Protection/detection: military working dog suitability test (*T*-test) success	GSD	14 months	71	Ratings by puppy raisers (of behavior over months)	Trainability (+), hyperactivity/restlessness (+), chasing/following shadows (+), stranger-directed fear (−), non-social fear (−), dog-directed fear (−), touch sensitivity (−)
E	Foyer et al.	2016	(96)	Protection/detection: military working dog approval for further training as decided by suitability test (*T*-test) leader	GSD	15–19 months	85	Behavioral assessment	Ambivalent and overt fear-related behavior (+)
F	Brady et al.	2018	(61)	Protection/detection: military working dogs and police dogs long-term success	GSD, LR, ESS, BM, DH, and crosses	4–5 years	79	Ratings by handlers (of behavior over months)	Responsiveness (+), energy and interest (+)
b	Wilsson and Sundgren	1997	(71)	Protection: police dog qualification	LR and GSD	450–600 days	2,107	Behavioral assessment	Courage (+), hardness (+), prey drive (+), defense drive (+), nerve stability (+)
G	Slabbert and Odendaal	1999	(97)	Protection: police patrol dog qualification	GSD	6 and 9 months	167	Behavioral assessment	Aggression (+)
B	Sinn et al.	2010	(58)	Protection: military working dog patrol-only certification	GSD, BM, and DS	1–3 years	1,000	Behavioral assessment	Search focus (+), sharpness (+), frontal bite (+), search stamina (+), static object interest (+)

*BMD, Bernese Mountain Dog; BM, Belgian Malinois; BT, Belgian Turvuren; DH, Dutch Herder; DS, Dutch Shepherd; ECS, English Cocker Spaniel; ESS, English Springer Spaniel; GR, Golden retriever; GSD, German Shepherd Dog; GWPX, Labrador-German wirehaired pointer cross; LBX, Labrador-Bernese Mountain Dog cross; LGX, Labrador-Golden retriever cross; LR, Labrador retriever; RP, Royal Poodle; RS, Russian Spaniel; SP, Saint-Pierre; V, Vizsla*.

##### Behavioral Measurements

Across these studies, behavior was measured in two main ways. The first method, labeled in Table 1 as behavioral assessment, refers to experimental approaches in which dogs were presented with a set of tasks, situations, and stimuli while their behavior was coded or scored by a trained rater in a standardized way. One example of this sort of assessment is the Dog Mentality Assessment (DMA), designed by the Swedish Working Dog Association, which consists of nine subtests involving social encounters, play opportunities, unexpected events, and work-related scenarios such as searching and protection [e.g., (94)]. Another example is the In-For-Training (IFT) test, often used by assistance dog schools, which exposes a dog to six potentially stressful scenarios, including a looming object, a sudden noise, and a threatening stranger, in order to gauge the dog's initial reaction and subsequent recovery [e.g., (79, 83)]. While the majority of test batteries, like the DMA and IFT test, focus on temperament traits, it is also becoming increasingly common to track the cognitive abilities of dogs, as in the Dog Cognition Test Battery [e.g., (82, 91)]. These sorts of empirical evaluations of temperament and cognition are helpful because they can be objectively scored by a small number of trained observers and the standardized format allows for direct quantitative comparison between individuals. Furthermore, in many cases, results from these tests are robust to variation in scoring methodology and are reliable whether an evaluator codes discrete behaviors or assigns an aggregate rating (46, 85, 86). However, because these assessments are often administered at just one or two timepoints, they provide a “snapshot” that may not be representative of the dog's behavior in other contexts or points in time. Furthermore, these tests can be quite labor and time intensive to administer.

The second method of measuring behavior involves ratings by puppy raisers or trainers that reflect the subjective impressions, formed over a period of weeks or months, of someone who has spent a lot of time with the dog. Rather than watching a dog encounter different scenarios in real-time, the evaluator reflects on the dog's typical response to a variety of situations when filling out a questionnaire. An example of this approach is the Canine Behavioral Assessment & Research Questionnaire [C-BARQ; (98)], a survey that is usually completed by the puppy raisers of assistance dogs at 6 and 12 months, but can also be completed by the handlers of adult dogs. It asks the respondent about the frequency or severity of behaviors that fall into multiple categories, including aggression, fear, attachment, excitability, and trainability [e.g., (62, 79, 83, 95)]. Another example of this type of instrument is the Dog Impulsivity Assessment Scale [DIAS; (99)], an 18-item questionnaire that requires the respondent to indicate level of agreement on items about behavioral regulation, aggression, response to novelty, and overall responsiveness [e.g., (61)]. Questionnaire surveys are advantageous in that they allow for information-gathering on a large number of dogs in a short amount of time. Furthermore, each evaluator has extensive knowledge of the dog's temperament, preferences, and habits, accrued by watching the dog navigate many different real-world environments and circumstances on repeated occasions. However, having so many different evaluators can have its drawbacks as well; evaluators receive no training, what might constitute high levels of a behavior to one person might seem inconsequential to another, and evaluators may not always have sufficient contact or context to make accurate assessments. Thus, a handful of studies have benefitted by using data from both behavioral assessments and questionnaires in their predictive modeling (79, 80, 83).

##### Behavioral Traits Associated With Working Dog Outcomes

**Figure 1:**
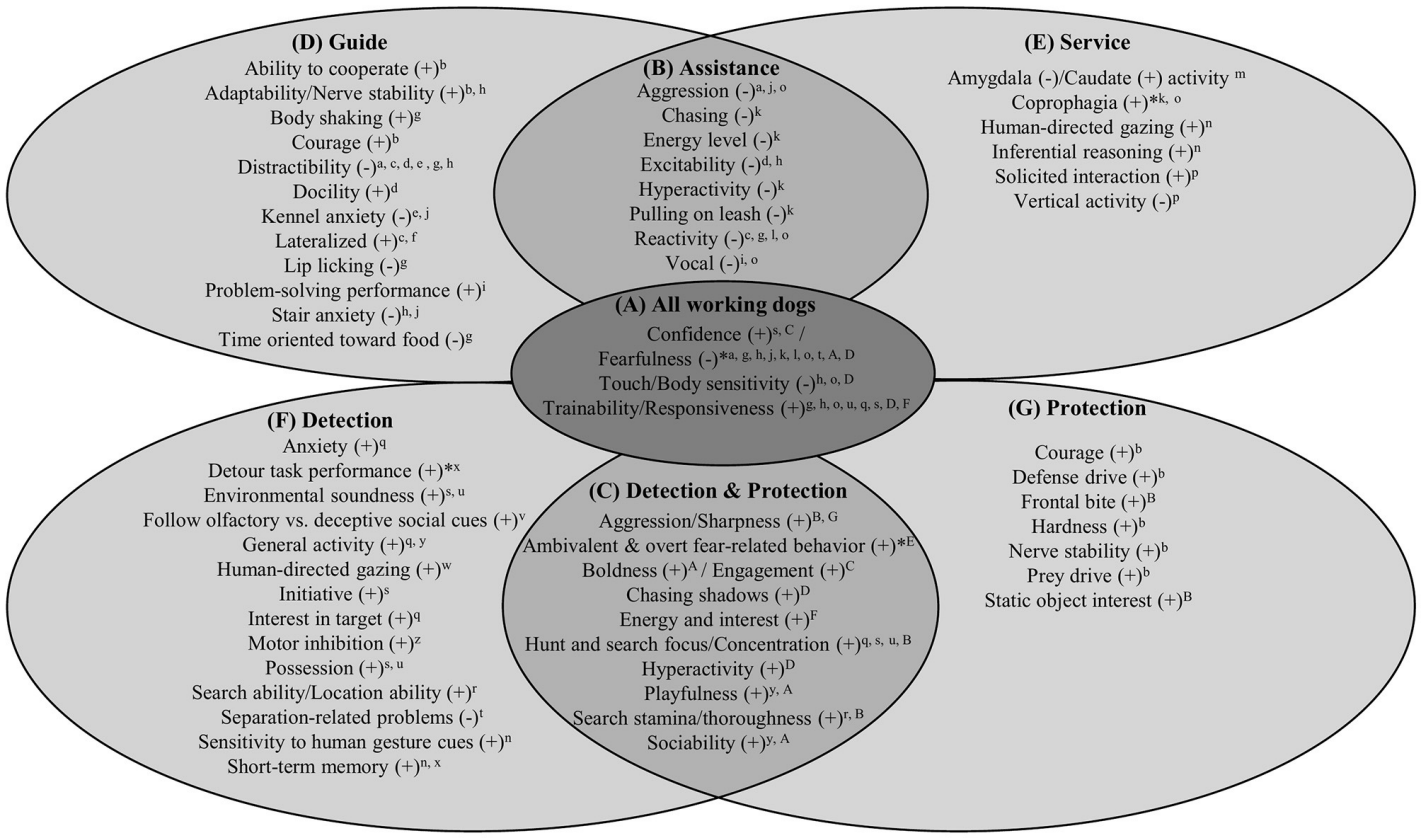
Behavioral traits implicated in the literature as associated with working dog outcomes. Here, we list the traits that have either been positively (+) or negatively (–) linked to successful working dog outcomes, categorized in the following ways: **(A)** traits common to all working dogs within the scope of this paper, **(B)** traits common to all assistance dogs, **(C)** traits common to all detection and/or protection dogs, as well as distinct traits unique to **(D)** guide, **(E)** service, **(F)** detection, and **(G)** protection dogs. These lists of traits are based on findings from the 33 empirical studies described in Table 1, and we have preserved the terminology used in the original papers. The relevant papers are referenced in superscript next to each finding, using the letters assigned to each paper in the first column of Table 1. It is worth noting that these findings are merely a reflection of the current literature; therefore, it could be the case that some of the traits that are only listed as important for guide dogs might also be important in service dogs, but the association has yet to be explicitly tested. In rare cases, the empirical studies supported contradictory results, and in those instances the disagreements are indicated with an asterisk.

In reviewing these studies, some common themes emerge regarding both desirable and undesirable behavioral traits in a candidate working dog (Figure 1A). For example, regardless of specific career path, multiple studies supported the notion that successful working dogs are highly trainable. Trainability and responsiveness was assessed using trainer ratings (84, 86), behavioral tasks (76), and questionnaires like the CBARQ (83, 95), puppy training supervisor questionnaire [PTSQ; (77)], and DIAS (99), which include multiple items asking the respondent to evaluate the dog's propensity to follow commands, learn new tasks, play fetch, attend to relevant stimuli, ignore distracting stimuli, and respond to correction. Additionally, using a measure of trainability based on an expert observer's assessment of ease and speed of learning new tasks, Lazarowski et al. (88) found that detection dogs specializing in alerting to person-borne explosives scored significantly higher than standard explosives detection dogs. Other important traits across all working dog categories included those which facilitate a steady, positive response to the environment: successful working dogs routinely displayed confidence or an absence of fear (46, 86, 94), whereas unsuccessful dogs tended to be more anxious, and fearful of dogs, strangers, and non-social stimuli (49, 62, 76, 77, 79, 80, 83, 87, 95). The one exception to this was a study which found that Military Working Dogs who evaluators approved for further training displayed higher levels of fear-related behavior, such as barking, support-seeking, and active avoidance, than non-approved dogs (96). Finally, unsuccessful candidates were also more likely to exhibit body and touch sensitivity—i.e., uncomfortable and tense reactions when being physically handled during events like grooming or physical examinations (77, 83, 95).

The literature also identified a suite of traits associated specifically with assistance dog outcomes (i.e., that apply to both guide and service dogs; Figure 1B). For example, multiple studies indicated that aggression, whether evaluated by puppy raisers (83), trainers (49), or both (79), was negatively linked to success. Opportunistically chasing small animals (62) and displaying high levels of reactivity, measured across scenarios involving exposure to sudden loud noises, prey-like objects, and unfamiliar stimuli (72, 76, 80, 83), were also predictive of poor outcomes. Relatedly, dogs rated as having high levels of energy, excitability, and hyperactivity were less likely to be placed as assistance dogs (62, 73, 77). In particular, behavioral manifestations of these traits—specifically, pulling on the leash (62) and inappropriate vocalizing (78, 83)—were associated with disqualification from assistance dog programs. Taken together, these findings paint a picture of an ideal assistance dog who is relatively quiet, calm, and unobtrusive. These findings are intuitive given that, whether picking up a dropped credit card or steering their handler around a pothole, both service and guide dogs frequently work in public settings where they must exhibit socially acceptable behavior.

Whereas guide and service dog populations share certain qualities that are either coveted or problematic with respect to their success, there are other features that they do not necessarily hold in common. For guide dogs specifically (Figure 1D), exhibiting high levels of distraction, and especially distraction with regards to other dogs, has been repeatedly identified as a trait that is detrimental to successful placement (49, 72–74, 76, 77). Furthermore, dogs who were visibly anxious around stairs (77, 79) or in training kennels (74, 79) were also less likely to graduate as guides. Finally, one study found that guide dog candidates who performed poorly and engaged in perseverative behaviors on a multistep problem-solving task were more likely to be released from the program, providing the first evidence that variation in cognitive skills can hold valuable clues about working dog potential (78). In terms of favorable qualities, successful guides ranked high on ability to cooperate—i.e., willingness to please (71), docility—i.e., high levels of tractability and learning ability (73), courage—i.e., once frightened, the ability to overcome that fear (71), and adaptability or nerve stability—i.e., ability to concentrate in high-stress situations but remain calm in frightening scenarios (71, 77). Interestingly, strength and directionality of paw preference (believed to be a proxy for lateralization in the brain) were also linked to guide dog success (72, 75). One potential explanation for this finding is an association between motor laterality and fearfulness, which has been indicated by past studies (100–102). Taken together, studies suggest that being able to effectively focus attention and problem solve, as well as exhibiting an adaptable attitude, are key factors to guide dog success.

In terms of behavioral traits particular to service dogs (Figure 1E), Weiss (63) found that dogs who displayed high levels of panic behavior—operationalized as vertical activity (i.e., rearing up) when left alone in an empty room for a short time—were more likely to be rated as having low service dog potential. Intriguingly, Bray et al. (83) also found that coprophagic dogs were more likely to graduate as service dogs; however, Duffy and Serpell (62) found no difference in coprophagic tendencies between successful and released dogs when looking across five guide and service dog organizations. Finally, recent research has also identified cognitive abilities that appear to be useful to service dogs. Graduate dogs consistently displayed higher levels of social looking across multiple experimental contexts, including a social referencing task and an unsolvable task (82). Given that service dogs must respond to human-given commands, these results are consistent with the type of behavior that is expected from a service dog in performing their day-to-day duties. Furthermore, using awake fMRI, Berns et al. (81) determined that certain brain activity patterns observed while the dog was viewing trained hand signals, including increased caudate activity and decreased amygdala activity, predicted success in a service dog program. Thus, for service dogs, a calm temperament and a strong propensity to direct attention toward humans appear to be beneficial.

In evaluating studies of dogs who engage in protection and/or detection work, a set of different (and sometimes opposite) characteristics materialized as important when compared to assistance dogs (Figure 1C). First, these dogs embodied a much more active phenotype: successful dogs scored higher on C-BARQ items indicating that they were hyperactive and had difficulties settling down, as well as prone to chasing shadows or light spots (95). Additionally, active military dogs and police dogs from multiple countries routinely showed high levels of energy and interest in their surroundings (61), as evaluated through the Positive and Negative Affect Scale (103). Successful military working dogs were also more bold, meaning they were sociable toward a stranger, playful during tug-of-war, chase-prone when presented with a moving object, and curious about startling events (94). They were also generally more willing to engage with their social and physical environment (46). Playfulness and sociability were also found to be associated with quicker drug detection times in a population of detection dogs, but only for German Shepherd dogs (92). Furthermore, unlike in assistance dogs, aggression was a desirable trait for detection and protection jobs; dogs who exhibited aggression and “sharpness”—i.e., a willingness to respond aggressively—in early adolescence were more likely to become police (97) or dual-certified military working (58) dogs. Unsurprisingly, successful protection and/or detection dogs also had a propensity to excel with search-related skills (85, 86, 88). For example, a high score on search focus—i.e., the ability for sustained, independent, olfaction-driven investigation without fatigue—was associated with both patrol-only and dual-certified qualifications (58). Overall, exemplary protection and detection working dogs appear to be energetic and assertive, and exhibit focused and thorough searching.

As a group, detection dogs also exhibited specific temperamental and cognitive phenotypes, as well as functional abilities related to their job (Figure 1F). High levels of activity, whether measured through a behavioral test (92) or trainer ratings (84), were linked to positive outcomes in drug detection dogs. In contrast to guide dogs, heightened anxiety was also indicated to be beneficial to drug detection dogs (84). Anxiety loaded together with other traits, including general activity, onto a component labeled “desire for work” that was associated with qualification (84). However, in search-and-rescue dogs in particular, separation-related anxiety was a negative trait; lower instances of separation-related problems were associated with FEMA-certification (87). Finally, multiple studies linked successful outcomes to the dogs ability to comfortably and adaptively respond to their physical and social environment, referred to as environmental soundness (86, 88) or initiative (86). In terms of cognitive skills, several studies provide evidence of the importance of social cognition. When presented with an unsolvable task at 11 months of age, dogs who eventually went on to qualify as detection dogs spent more time gazing toward the human (90). Furthermore, the ability to follow a human communicative gesture in a cooperative food-finding context was also linked to explosive detection dog success (82). Crucially, however, when olfactory and social cues were pitted against one another, the tendency to make choices guided by olfactory cues over misleading social cues predicted detection dog success (89). Cognitive skills outside the social realm were also significant; positive detection outcomes were linked to increased short-term memory (82, 91), better motor inhibition (93), more correct choices during the acquisition phase of a detour task, and longer latencies to detour during the first reversal trial of that same detour task (91). There were also several job-specific behaviors that differentiated detection dogs who qualified from those who did not. Successful detection dogs were extremely interested in an object doused in scent (84), possessive of objects (86, 88), and quick to locate hidden explosives without assistance from the handler (85).

Finally, the literature identifies several traits which appear to be advantageous in protection dogs (Figure 1G). Similar to guide dogs, successful protection dogs exhibited high levels of courage and nerve stability, meaning that they reacted appropriately and were resilient and focused when faced with high-stress or frightening scenarios (71). Additionally, effective police dogs showed high levels of “hardness,” indicating that neither corrections nor frightening experiences affected them strongly (71). They also displayed other traits which are related specifically to the function of protection dogs. For example, patrol certification was more likely for dogs who exhibited a strong frontal bite and extreme interest in a rubber toy (58). Relatedly, police dogs showed an especially high drive to engage in competitive games—i.e,. prey drive—and a tendency to defend themselves and their handlers—i.e., defense drive (71).

Although the studies reviewed here are highly informative about relevant adolescent and adult behavior, it is widely acknowledged that the earlier an accurate prediction can be made about the ultimate suitability of a potential working dog, the better. Thus, there is longstanding interest in aptitude testing with puppies. However, evidence regarding whether adult characteristics can be accurately predicted from those of puppies remains mixed. While there are several studies that find evidence for long-term stability in temperament (76, 77, 104–109), others find little evidence for these associations (110, 111). Furthermore, while cognitive traits have been much less studied, there is emerging evidence that some traits, such as those related to executive function, social communicative skills, and odor discrimination, show moderate stability over time, while others, such as memory and auditory discrimination, do not (91, 112). Finally, while multiple studies have found that early screening (i.e., 12 weeks or younger) of puppy temperament is not very effective in predicting working dog success (104, 113, 114), there are others that suggest assessments of puppy behavior do have some predictive value (97, 106, 107). Given their potential utility, and the fact that we continue to refine our understanding of which traits are most important, more research into the predictive value of puppy testing is clearly warranted.

The studies reviewed above identify aspects of behavior and cognition that are associated with working dog success; however, the causes of variation in these traits remain poorly understood. Given the complex nature of most behavioral and cognitive processes, we expect that these traits will be influenced by both environmental and genetic factors. Although knowledge about these processes remains limited, we review key findings about environmental and genetic associations with working dog success in the following two sections.

#### Environmental Factors

Early environmental experiences are known to have profound and lifelong effects in many animals. For example, in rodents and primates, the amount and type of maternal care experienced by infants has wide-ranging effects on later development. Extreme disruptions in early maternal care (e.g., 1–3 h separation over multiple days, or a single 24-h separation period) adversely impact later offspring cognition (115–117), whereas shorter separations from the mother and social group appear to have inoculating effects, dampening stress responses (118) and enhancing cognition (119, 120). Importantly, even natural variation in the quantity and quality of maternal care that offspring experience over early development has been demonstrated to have long-lasting effects on later stress responses (121), behavior (122, 123), and cognition (124, 125).

Recently, it has been suggested that the same is true in dogs, with maternal care posited to play a crucial role in the behavioral development of puppies (126–128). As we learn more about the optimal maternal conditions for working dogs (see section 1.2.1 below), people who procure dogs should consider the early environmental conditions that candidate dogs experienced. While more research is needed, it seems clear that an objective measure of maternal care could be one useful future metric when predicting a dog's working potential (78).

It is also likely that experiences during the juvenile period, lasting from ~12 weeks to 6 months, are similarly formative. Unfortunately, canine research covering this time period is scarce (129). However, the few studies that have been conducted in working dogs provide support for the notion that the environment over this period has important impacts on behavior in adulthood (130). For example, Serpell and Duffy (131) surveyed the puppy raisers of over 975 prospective guide dogs about their dog's behavior as well as features of the dog's environment. They found significant effects of many aspects of the home rearing environment on dog behavior measured at 12 months of age. Living with a more experienced puppy raiser (quantified as number of trainee guide dogs previously raised) was associated with less aggression toward people and dogs, as well as lower levels of dog-directed fear, non-social fear, and touch sensitivity. Being raised in a household with other dogs was also associated with less aggression toward household members. Finally, reported traumatic events during the juvenile period had significant effects on later expression of defensive behaviors, with dogs who were attacked or threatened by an unfamiliar dog exhibiting higher levels of dog-directed fear and stranger-directed aggression, and dogs who were frightened by a person exhibiting high levels of stranger-directed fear. In a similar study of a different guide dog population, Harvey et al. (132) also found effects of puppy raiser experience and the social environment. Puppy raiser experience was associated with lower levels of energy and distractibility. In terms of the social environment, being raised in a household with other dogs and children was associated with higher energy levels, excitability, and trainability. Additionally, dogs who were given more opportunities to play with other dogs scored lower on separation-related behaviors. Importantly, many of these behaviors are in turn associated with working dog outcomes.

And in fact, two studies have linked certain aspects of juvenile dogs' experiences directly to working dog outcomes. Foyer et al. (95) found that trainee military working dogs who were left home alone for longer periods during the day were subsequently more successful. The authors note that, as this finding was correlational, it is likely the case that being left alone longer was more feasible in dogs who were more resistant to stress, a behavioral feature that is desirable in working dogs. Regardless of the mechanism, it reveals an easily monitored feature of the early environment that can be a useful indicator for future working dog outcomes. In guide dogs, Serpell and Duffy (131) found that experiencing a traumatic event—specifically, being frightened by a person—was significantly associated with a lower likelihood of becoming a guide, whereas being raised in a household with other dogs and pets was significantly associated with a higher likelihood of becoming a guide. Again, these are fairly straightforward measures that can easily be reported by the puppy raiser.

#### Genetic Factors

Although there remains much to be learned about the genetic bases of complex traits in dogs, it is already clear that many behavioral traits critical to working dog success are strongly influenced by genetic factors (114, 133–136). As described below (see section 1.2.2), this knowledge has important applications in the context of breeding dogs for working roles. However, genetic factors can also be considered when attempting to identify dogs with potential for success. Here, it is important to distinguish between approaches based in quantitative and molecular genetics (137). Quantitative genetic approaches make use of knowledge regarding the heritability of particular traits, and relatedness between individuals in a population. Estimated breeding values (EBVs) reflect an animal's genetic merit with respect to a phenotype of interest and incorporate the heritability of this trait. When candidate dogs are selected from a population in which phenotypes, relatedness, and heritability of key traits are known, EBVs provide a useful measure for identifying dogs with the most genetic potential for success. Using molecular genetics, it is also possible to estimate an animal's phenotypic potential using marker-assisted selection. As the name implies, this process relies on screening animals based on known genetic markers that are associated with the phenotype of interest. This method has advantages in that an animal's potential can be estimated directly from their genotype, and this approach has flourished in plant and production animal breeding (138). Although marker-assisted selection and successor approaches such as genomic selection—which makes use of variants throughout the genome—are expected to become increasingly common, at present they entail notable challenges in terms of implementation with dogs [(139), Karlsson et al., submitted this volume].

Nonetheless, early work has identified some genotypic markers that may be useful in the selection of working dogs. In general, domestic dogs have been found to be hyper-social compared to non-domesticated canids, which is one factor that likely facilitates their success living and working in human environments (140). Structural variants in *GTF2I* and *GTF2IRD1* genes have been linked to extreme sociability in dogs in general (140). However, individual variability between dogs also exists. Hyper-focus to social stimuli and heightened gregariousness is often considered desirable for some working roles, such as therapy and assistance dogs. On the other hand, it may also interfere with a dog's ability to cope when left alone or to focus on non-social stimuli, skills that are critical to independent problem-solving success central to other working roles (141). Therefore, in some cases genetic screening may provide an opportunity to match dogs with training or working opportunities that capitalize on their behavioral predispositions, and help them achieve their full potential. However, it is important to note that even in dogs predisposed to high sensitivity to social stimuli, best practices in socialization and training are still critical to the quality and success of social interactions with humans, in addition to the dog's well-being.

For detection and protection dogs, more preliminary molecular genetic approaches have been undertaken. For example, a variable number tandem repeat (VNTR) in a neurotransmitter-associated gene, Tyrosine Hydroxylase (*TH*), has been associated with impulsivity in dogs (142) and even the probability of success in a Korean military dog training program (143). Further, targeted candidate gene approaches have had preliminary success in identifying dogs with the greatest potential in Korean military dog programs (144, 145). Additionally, a candidate gene approach recently identified several single nucleotide polymorphisms (SNPs) associated with variable canine olfactory detection performance (146, 147), suggesting that a molecular genetics approach may be fruitful in identifying detection dogs with higher performance capabilities. However, more research and application to other populations of working dogs is necessary before robust conclusions can be drawn or such a technology can be implemented as a selection tool.

#### Dogs Who Choose Their Own Jobs: Accounting for the Preferences and Skill Set of the Dog

Most programs that raise and train working dogs are focused on a single career path, whether that is assistance or detection work. As a result, the success rate of the dogs in these programs is often limited [e.g., 50% or less; (62)]. An alternative approach is to identify the essential foundational skills that are required by all working dogs and then select for the dogs with those skills combined with general health characteristics. Then, the dogs can be exposed to basic training before undergoing reproducible and valid screening tests to determine their physical and behavioral strengths and preferences. For example, a future search-and-rescue dog needs to be comfortable working independently of the handler and moving confidently over unstable surfaces. If a dog does not have these natural tendencies, with training and a lot of effort, they may be able to achieve the basic skills to function as a search-and-rescue dog, but a dog that has natural tendencies for these skills will reduce the necessary training time and increase the likelihood of success. Additionally, from a welfare perspective, expressing natural behaviors is thought to be intrinsically rewarding to a dog (148). In order to maximize the success of working dogs and thoughtfully place each dog in a role that is suited to that dog's physical abilities and temperament, the phenotype associated with each career path needs to be clearly defined and tests validated to predict performance. This approach is analogous to the screening measures described above, but rather than initially screening for specific cognitive or behavioral characteristics, it instead allows dogs to first engage in basic components of a variety of working roles, providing important insights about natural proclivities for various components of these jobs.

### Improving and Cultivating Characteristics Linked to Working Dog Success

The studies reviewed above address methods for potentially identifying dogs with high potential for success, which can be considered as a type of “aptitude testing.” However, a complementary approach involves active intervention to cultivate desirable phenotypes. This process can be effectively implemented at two distinct levels. First, at the individual level, we can intervene over the course of dogs' lives to set them up for success in several ways, including manipulating their early environment, promoting healthy habits, and intentionally fostering desirable qualities. For maximum efficacy, these approaches require early access to the dogs who will ultimately be trained for working roles. Second, at the population level, we can intervene over generations to strengthen future populations of dogs through genetic selection of heritable traits. This second approach will be most feasible for programs that breed their own dogs or function as part of a breeding cooperative, as it requires access to pedigrees and the ability to estimate heritability of various traits within a particular population.

It is worth noting that intervention at both the individual and population level necessitates a clear understanding of which traits are desirable for each of the career options. Below, we review the best practices as suggested by the literature. However, as our understanding of which characteristics contribute to working dog success continues to grow and evolve, that will directly inform the environmental features, physical characteristics, and behavioral traits that should be targeted for improvement. Furthermore, some suggestions will apply to characteristics that benefit all working dogs—for example, all working dogs need healthy hips, whether they are an assistance dog or a detection dog—whereas others will be specific to certain roles; for example, the ability to effectively use olfaction is crucial for a detection dog but largely irrelevant for most assistance dogs.

#### Modifications at the Individual Level: Evidence-Based Improvements in Rearing and Training Practices

##### Early Environment

In recent years, several studies have reported associations between early maternal environment and later offspring behavior. For example, after surveying the families of over 3,000 Finnish dogs, Tiira and Lohi (149) found, based on owner-reported measures, that dogs who experienced lower levels of maternal care displayed higher levels of fearfulness as adults. In an observational study in which Beagle dogs whelped in a professional center, researchers coded maternal behavior and found that puppies who experienced higher levels of maternal care over the first 3 weeks of life were more exploratory and showed lower levels of anxious behaviors, such as vocalizations and increased movement, during a 3-min isolation task administered at 2 months of age (150). Interestingly, a second study by the same group observed pet dogs of various breeds who whelped in family homes and found essentially the opposite result, wherein higher levels of maternal care were associated with more stress behaviors and less exploration and play in the eight-week-old puppies (151). These differences raise interesting questions about how the expression of maternal care potentially differs between populations of dogs, as well as how maternal care interacts with other aspects of the early environment to affect offspring characteristics.

While these studies were not conducted in working dogs, the behaviors found to be associated with maternal care have substantial overlap with those perceived as important to working dog roles. Furthermore, in a study that did specifically examine military working dogs, Foyer et al. (152) found an association between maternal behavior and later puppy behavior over 1 year later—-German Shepherd dogs with more involved mothers were more interested in people, more comfortable in novel environments (e.g., on metal stairs or shakey tables), and more aggressive. Bray et al. (78) conducted a study in a population of guide dogs and found that high levels of maternal behavior were linked to stress and anxiety in the offspring as adolescents (i.e., higher activity levels in an isolation task and a reduced latency to vocalize during a novel object task), as well as worse performance on a problem-solving task. This same study also directly linked maternal style, experienced by the puppies over the first few weeks of life, to working dog outcomes as adults. Puppies whose mothers displayed higher levels of maternal care, operationalized as nursing, licking/grooming, contact, and proximity (153), were less likely to succeed as guide dogs up to 2 years later. Furthermore, puppies whose mothers more often nursed from a sitting or standing position were more likely to graduate as guide dogs. One potential explanation for these findings is that small doses of mild stressors—i.e., having an adequate but less responsive mother, nursing from a more challenging position—might help to facilitate resilience from a young age. This idea of mild stressors leading to positive long-term outcomes is echoed in the handling literature, wherein introducing brief separations from the mother and handling (i.e., tactile stimulation) by a human in the first few weeks of life has been associated with positive emotional and cognitive outcomes in both rodents (154–157) and dog puppies (158–160). Thus, especially as we continue to learn more about the long-ranging effects of early environment over the first few weeks in dogs, breeders can use these findings to encourage and generate optimal conditions.

Additionally, the timing and circumstances under which puppies are introduced and acclimated to social stimuli, such as people, along with a diverse sampling of environmental features, are critical. For example, it is well-established that experiences during the socialization period, which encompasses the time window from roughly 2.5 to 14 weeks (129), can have major implications for later behavior (161). In working dogs specifically, guide dog puppies that were whelped in a kennel and first integrated into a home environment at 12 weeks of age were significantly more likely to later graduate as guides than puppies who did not leave the kennels until 13 weeks of age or later (162). However, the ideal weaning time, especially as it pertains to working dogs, is both understudied and debated in the literature (129). On the one hand, 6–8 weeks has been identified as the best time to start building the human-canine bond and begin gaining exposure to aspects of the later working environment (163, 164), and yet there is also evidence that weaning too early can be overly stressful, adversely affecting health and behavior (165–167).

As puppies develop, proper socialization through exposure to varied stimuli, ideally prior to 14 weeks of age, is key to the dog becoming a well-adjusted and resilient adult (54). Early, consistent experience with speech and music via radio clips (168) and video images (169) during the first few weeks of life have been linked to decreased noise reactivity and neophobia, respectively, around 7–8 weeks of age. Relatedly, a lack of exposure to urban environments between 3 and 6 months of age was associated with aggressive and avoidant behaviors (170). There is also evidence in military working dogs that increasing their amount of human contact—i.e., housing dogs with their handler instead of kenneling, implementing a socialization program—is associated with decreased fear and aggression (171, 172). However, while experts agree that exposing puppies to all of the environmental features that they will encounter over the course of their job is essential, this exposure should be done in an intentional, controlled way. For example, when first introducing dogs to specific fear-eliciting stimuli, optimal responses were obtained when dogs were first given the chance to habituate (173). Furthermore, the social context during exposure to novel and/or potentially scary stimuli is important to consider; when a dog is given the opportunity to observe how conspecifics and/or humans react to a given situation, it can in turn inform how the dog reacts, either positively or negatively (54, 174).

##### Physical Soundness

Physical soundness is based on the structure and physical conditioning of the dog. Much of the structure will be a function of genetics. Dogs with physical limitations such as heart conditions, respiratory compromise from brachycephalic syndrome, and/or musculoskeletal or sensory anomalies are unlikely to be effective working dogs. Physical conditioning provides an opportunity to enhance the dog's health, behavior, and longevity. However, implementation of a formal exercise program for puppies has been subject to controversy, and there is currently little data to support definitive recommendations. Most of what is known about exercise during development is derived from animal research studies or human reports (175–177). The primary concern about early introduction of exercise is related to the potential effect of repetitive motion on bone growth and joint development. One observational study of puppies <3 months of age reported an increase in hip dysplasia in puppies with access to stairs (176, 178), although the same study reported a decreased risk in puppies with access to off-leash exercise. In an intensive treadmill study of young Beagles, the bones and joints of the limbs were not adversely affected; however, early evidence of osteoarthritis was detected in the spine (179). Most studies find there are benefits to moderate exercise during development, including mental stimulation, improved muscle development, joint stability, bone development, and coordination. Physical activity and the associated increase in muscle strength and joint stability as well as decreased body fat leads to a decrease in the development of osteoarthritis (180, 181). Continuation of an exercise program throughout the life of the dog is likely to decrease injury and improve recovery from injury. A recent foundational fitness program for working dogs has been proposed for working dog puppies through adults (182). This Fit to Work program focuses on mobility, stability, strength, and proprioception and avoids repetitive high impact activities. As a foundational program, it does not address job-specific physical requirements or cardiovascular stamina, which should be tailored for individual careers. Finally, in addition to the physical benefits, daily exercise is linked to a decrease in both noise sensitivity and separation anxiety (149), as well as improved quality of life scores (183). Regardless of the age of the dog or the career path, some component of physical exercise is essential for the physical and behavioral health of working dogs.

##### Behavior (Temperament and Cognition)

To foster desirable behavior, one technique which organizations could implement is to work with puppy raisers to more systematically track cognitive and temperamental tendencies of interest in each dog from an early age, and to then provide tailored advice and support (184). By doing so, potential red flags could be identified earlier and then actively addressed during rearing and training. Additionally, decisions could be made earlier about which working dog path might be the right fit. One way of achieving this goal is through prescriptive (as opposed to predictive) testing. An example of this sort of practical diagnostic tool that has recently been developed for pet dogs is the AKC Temperament Test [ATT; (185)], which evaluates a dog on a set of standardized tasks that allow problem behaviors to be identified. As part of the testing, concrete training materials are then provided in order to help the raiser modify any problematic behaviors. In fact, evidence-based training techniques are likely to be instrumental in influencing the behavior and subsequent success of working dogs, during the puppy raising phase but especially during the professional training phase. For a detailed discussion of best practices in working dog training, please see Hall et al. (submitted, this volume).

In some cases, dogs may work part-time and live as pets with their owner or handler when not working—for example, this is true for many volunteer search-and-rescue dogs (42) as well as therapy dogs (186). It is also true that not all dogs who serve in a working role were specifically bred or selected for that purpose initially. For example, a dog may become an assistance animal for their owner, or another individual in the home, after living primarily as a pet in that same household previously. Even in cases where dogs were bred specifically for a working role (i.e., an individual bred and raised to be a guide dog), the connection shared between the dog-human pair often extends beyond that of a working relationship. In all of these cases, considering the quality of the dog-human bond may be an important and relevant factor for predicting the dog's work performance, as well as the well-being of both the dog and human involved. A growing area of research is focused on attachment bonds developed between dogs and humans (187–189) and is relevant given that attachment security is broadly associated with stress reduction, resilience, comfort in novel situations and environments, and exploration and learning in both humans and dogs (190, 191). There is considerable research demonstrating that how a human perceives the quality of the relationship they share with a dog—for example, how attached they are to the dog they own—can have a significant impact on human health and well-being (189, 192). Several studies have also documented the benefits (e.g., increased well-being and job satisfaction) of a close relationship to the dog with whom the handler works across animal assisted therapy (192), assistance dog (193), and protection/detection dog (43) contexts.

Less research has focused on the impact of a dog's attachment security toward their owner/handler on their own well-being and performance. However, the research that has been done has suggested that working dogs with a secure attachment to their handlers exhibit more resilience and faster stress reduction in novel environments (191, 194, 195), may perform better in therapeutic contexts (191, 196), and are more persistent at problem solving (190) when compared to dogs with insecure attachment bonds. Dogs are also known to form attachment bonds to new humans rapidly (197, 198), even into adulthood (199). Furthermore, research suggests other, related aspects of working dog-handler relationships are associated with overall performance; avalanche search dogs who were confident working farther from their handler were more successful (200), and detection dogs with higher levels of familiarity with their handlers were more effective (172, 201, 202), as well as less fearful and aggressive (171). When surveyed, Transportation Security Administration (TSA) detection dog handlers reported that they viewed the relationship to the handler as one of the most important traits for work; however, it is not currently accounted for in the organization's behavioral tests (70). A more thorough investigation of how relationship quality between dogs and their owners, handlers, trainers, and other humans they encounter in their working role impacts performance and welfare is an important area for additional future research. However, the current research suggests that taking the time to foster a strong bond and familiarity with the dog's working partner is one avenue through which to improve working dog performance.

By facilitating healthy, happy, and resilient puppies from the first few weeks and continuing across development, the hope is that these dogs will be better prepared to face the cognitive and temperament challenges awaiting them on the job, ultimately resulting in lower attrition rates from working dog programs.

#### Modifications at the Population Level: Breeding for Working Roles

The practice of breeding for particular characteristics in dogs has ancient origins and is responsible for the extraordinary phenotypic variation among modern breeds (134, 140, 203, 204). Because many working dog organizations maintain (and share) dedicated breeding populations, there is great potential for continual improvements in these populations through selective breeding. However, it is important to note that the effectiveness of this approach relies on both an understanding of the genetic and environmental influences on particular traits, as well as reproducible methods for measuring phenotypes of interest (139). Heritability—i.e., the proportion of variance in a trait that is due to additive genetic factors—is a key determinant of the potential for selective breeding. Highly heritable traits respond strongly to selection, thereby providing opportunities for rapid improvements in a population. In contrast, traits with minimal heritability present less attractive targets for breeding, and in the case of traits with no heritability, selective breeding is futile. Below, we highlight several opportunities and areas of progress in the breeding of better working dogs.

##### Physical Soundness

With respect to physical health, appropriate health screening of working dogs is critical prior to selecting breeding pairs or working dog candidates. A complete medical evaluation includes an eye examination, cardiac auscultation, and ultrasound if indicated. Genetic diseases known to affect the breeds being used (e.g., degenerative myelopathy in German Shepherd dogs, exercise-induced collapse in Labrador retrievers) should be cleared by heritage or by testing. Mobility is required for all dogs and essential for working dogs. One of the most common causes of early retirement in military working dogs is spinal cord disease and arthritis (60, 180, 182, 205).

Although phenotypic screening can be important in breeding decisions, phenotypes of individual animals arise through a combination of genetic and environmental factors. More direct approaches to identifying genetic merit use estimated breeding values (EBVs), which can be informed not only by the characteristics of an individual dog, but also by their relatives, and knowledge of the heritability of the trait(s) in question, while adjusting for environmental effects. The use of EBVs, which is widespread in production animals (206), has become more common in dog populations in recent decades (207). For EBVs to be most effective, it is crucial to have values on phenotypes of interest from as many members of the population as possible (i.e., not just the potential breeding dogs, but also their siblings, parents, and grandparents). These measures have been used successfully for genetic improvements in hip and elbow dysplasia, which are conditions that lead to secondary osteoarthritis, compromise dog welfare, and can shorten the livelihoods of working dogs. Due to the relatively high heritability of these traits (47), some working dog organizations have drastically reduced their incidence through selective breeding programs. For example, in a study across three different breeds, the percentage of dogs with “excellent” hip scores was increased from 34–55% to 87–94% within eight generations of selection (208).

##### Behavior (Temperament and Cognition)

Breeding for behavioral traits has proven to be more difficult, in part due to the challenges of large-scale adoption of standardized phenotyping procedures. Currently the genetic contribution to many behavioral traits remains poorly understood, but heritability studies are becoming increasingly common (71, 133, 134, 136, 209–214). To our knowledge, most working dog organizations that currently incorporate behavioral measures in EBVs rely on organization-specific phenotyping procedures which are often tailored to specific working roles. Thus, unlike measures of disease susceptibility, which rely on highly standardized phenotypes with common value across working roles, behavior presents somewhat of a “moving target” which may be quantified and valued differently across organizations and working roles. Indeed, many of the most pressing challenges with respect to behavior involve simply identifying behaviors that predict working dog success. Only after these traits have been identified, and a heritable basis for them determined, can selective breeding move forward productively. Nonetheless, the potential to successfully select for behavioral traits conducive to working roles is suggested by the substantially higher probability of success for dogs from working dog breeding programs as opposed to outside stock of the same breeds (49, 59).

## Future Steps

The work reviewed above presents promising advances with respect to both our scientific understanding of working dogs, and the practices through which these dogs are selected, bred, and trained. In fact, many of the best practices discussed will also be relevant to other types of working dogs not explicitly discussed in this paper, as well as companion dogs. For example, being intentional about selection of breeding dogs, health and genetic screening, socialization, and cultivating the human-dog bond is crucial for producing healthy, well-adjusted dogs in general. However, specifically with regards to working dogs, we recognize important knowledge gaps and limitations that will be important to overcome in the future. Thus, we conclude by identifying key limitations of current research and practice and provide recommendations for future work in these areas.

### Defining and Understanding Working Dog Success and Failure

One key challenge relates to defining success as a working dog. In most studies, success has been defined primarily as completion of the associated training program and placement into a working role. While indeed a functionally relevant outcome, this measure is subject to several important limitations. First, organizations vary widely in the proportion of dogs who complete training programs and this variability arises in part due to differences in procedures, standards, and criteria across organizations. Large established organizations, who breed and train thousands of dogs per year, can afford to release dogs from training programs more so than smaller organizations who have access to limited numbers of dogs. Similarly, large organizations may be better positioned to simply release a dog from the program if this dog exhibits undesirable characteristics, rather than investing substantial effort toward trying to modify the dog's characteristics. Second, the extent to which various undesirable characteristics (e.g., minor medical problems, nuisance behaviors) are acceptable in a placement likely varies widely between programs as well. Thus, it is typically not meaningful to compare success rates between different organizations, and training success does not reflect any single set of objective criteria. Decisions to release a dog from a training program are typically made on a case-by-case basis, relying on the intuitions and judgements of training staff (rather than objective performance on a standardized evaluation). Although these decisions are made by subject-matter experts, they inherently involve a degree of subjectivity. To our knowledge, it is also rare that working dog organizations assess interrater agreement in these contexts, making it unclear to what extent independent evaluators would arrive at similar conclusions. Lastly, evaluating success based simply on completion of a training program fails to consider the extent to which a dog is ultimately able to carry out their duties once placed in the working environment.

From a practical perspective, key measures of a working dog's impact relate to the dog's ability to perform their role once placed in the working environment, as well as the longevity of successful performance in this context. Batt et al. (45) propose an important distinction between training success and working success. For example, in one large-scale study, nearly a fifth of guide dogs were withdrawn from their working roles due to behavioral problems (215), despite having successfully completed the initial training program. However, the causes of failure in these types of circumstances can be challenging to identify. Many working dogs function as a team together with a human handler, and these handlers may vary in their skill or compliance with activities required for the dog to function effectively. Thus, the ultimate success or failure of a dog often depends considerably on the handler(s) with whom they work. Nonetheless, it is important to recognize that robust definitions of success should incorporate measures of a dog's performance in the actual working role, not merely the prerequisite training. Ideally, working dogs should undergo regular evaluations in which they are assessed or recertified by a professional evaluator to ensure they remain fit for their roles.

Tolstoy famously began his novel Anna Karenina with the phrase that “all happy families are all alike; each unhappy family is unhappy in its own way” (216). Somewhat analogously, within working dog programs, successful dogs tend to share many of the same characteristics required for success, whereas the reasons for failure are myriad and complex. Although it is convenient in research to classify outcomes as success vs. failure, it is important to recognize that the latter category will almost always consist of dogs who were unsuccessful for different reasons. In most studies to date, common problem behaviors (e.g., excessive barking, separation anxiety, dog-directed aggression) have clear negative impacts on a dog's potential success. However, each of these behaviors may have a different and complex etiology making it challenging to identify unifying themes that are shared among unsuccessful dogs. Additionally, although the presence of these types of behaviors is decidedly negative, the mere lack of problem behaviors is typically not sufficient for success. This phenomenon is nicely illustrated through predictive modeling studies in which dogs with a history of problematic behaviors can be reliably identified as having a low probability of success, whereas the lack of these behavior problems does not necessarily translate to a high probability of success (83). Thus, it will be important to understand working dog potential both in terms of the absence of problematic tendencies as well as the presence of other favorable traits required for a specific working role (130). At present, researchers have been more successful in developing assessments of the former, making tools to achieve the latter an important priority for future work.

Based on the challenges reviewed above, both dog providers and researchers should strive to develop and implement outcome measures that go beyond simple definitions of success or failure, and instead quantify dogs' ultimate strengths and weaknesses across multiple functional domains. Ultimately, these outcome measures will provide important endpoints for selection tools that not only identify a dog's probability of success, but also identify the specific areas in which a dog is likely to excel or struggle. In turn, these approaches will inform interventional strategies that can be catered to the characteristics of individual dogs.

### Research Methods

Whereas studies of medical conditions and physical characteristics of working dogs have aligned on widely-used standardized assessments [e.g., hip scores; (217)], studies of working dog behavior and cognition tend to employ a greater diversity of methods. By far the most standardized behavioral measures involve survey-based assessments [e.g., CBARQ; (98)], which are relatively easy to administer identically across different populations and study designs. In contrast, experimental studies of working dog behavior and cognition are still in a period of active method development and validation, and there are few standardized research methods that have been adopted across the industry.

Although a lack of standardization can be viewed as a weakness, we argue that standardization for the mere sake of standardization does little to advance the field. Before aligning on standardized approaches, it is critical that the approaches being adopted are rigorously evaluated in terms of their validity and applicability to diverse populations and working roles. Currently many of the approaches described in this paper are in nascent phases of development and it will be important to allow them time to reach maturity before encouraging widespread adoption. During this time, communication between researchers and professionals in the field with years of hands-on working dog experience should be prioritized (64, 67, 70, 218). These conversations will help address the disconnect of researchers conducting tests that are then either not useful or not routinely used, as well as organizations running tests that have not been analyzed or validated. Crucially, as the field moves through this period of development, it is more important than ever that researchers employ rigorous experimental designs along with objective and transparent approaches to scoring and analysis, such that the products of this research can be adequately evaluated by both scientists and practitioners in the field (65). Along these lines, researchers should aim to [1] present methodological details such that all components of a study are fully reproducible, [2] conduct and report inter-rater reliability for measures used, [3] conduct and report retest reliability, [4] differentiate between approaches that describe statistical association vs. predictive validity, [5] employ well-powered designs that control for confounding factors, and [6] ensure adequate blinding between research staff and dog training professionals.

Lastly, it is important to emphasize that while we identify a range of opportunities for improving the performance of working dogs, due to diversity in working dog roles and the organizations in this arena, there are few one-size-fits-all solutions. Large established organizations who breed hundreds of dogs per year are undoubtedly in better positions to make use of selective breeding, EBVs, and potentially even genomic selection methods to improve their populations over time. Smaller organizations, and those who source dogs from more heterogeneous populations, stand to achieve larger gains through enhanced techniques for identifying individual dogs with high potential, as well as individualized interventions that would be challenging to implement at larger scales.

Although specific applications will vary across the industry, it is important to recognize that when considered collectively, the science of working dogs has enjoyed many notable advances throughout recent decades. We expect that continued collaboration between scientists and practitioners will play a critical role in the future of this enterprise, which has great potential for enhancing the health and well-being of both working dogs and the people they serve.

## Author Contributions

EB, NH, EM, CO, and MU contributed to writing—original draft. EB contributed to supervision and visualization (Figure 1 and Table 1). All authors contributed to conceptualization, investigation, funding acquisition, and writing—review & editing.

## Conflict of Interest

The authors declare that the research was conducted in the absence of any commercial or financial relationships that could be construed as a potential conflict of interest.

## Footnotes

^1^https://assistancedogsinternational.org.
