# Erratum: Enhancing the Selection and Performance of Working Dogs

**DOI:** 10.3389/fvets.2022.898698

**Published:** 2022-04-08

**Authors:** Frontiers Production Office

**Affiliations:** Frontiers Media SA, Lausanne, Switzerland

**Keywords:** assistance dogs, canine, detection dogs, selection, temperament, working dogs, protection dogs

Due to a production error, the references for “169” and “172” were incorrectly written as

“169. Pluijmakers JJTM, Jolanda JT, Appleby DL, Bradshaw JWS. Exposure to video images between 3 and 5 weeks of age decreases neophobia in domestic dogs. *Appl Animal Behav Sci*. (2010) 126:51–8. 10.1016/j.applanim.2010.05.006

172. Haverbeke A, Rzepa C, Depiereux E, Deroo J, Giffroy JM. Assessing efficiency of a human familiarisation and training programme on fearfulness and aggressiveness of military dogs. *Appl Animal Behav Sci*. (2010) 123:143–9. 10.1016/j.applanim.2009.12.014”

They should be

“169. Pluijmakers JJTM, Appleby DL, Bradshaw JWS. Exposure to video images between 3 and 5 weeks of age decreases neophobia in domestic dogs. *Appl Animal Behav Sci*. (2010) 126:51–8. 10.1016/j.applanim.2010.05.006

172. Haverbeke A, Rzepa C, Depiereux E, Deroo J, Giffroy JM, Diederich C. Assessing efficiency of a human familiarisation and training programme on fearfulness and aggressiveness of military dogs. *Appl Animal Behav Sci*. (2010) 123:143–9. 10.1016/j.applanim.2009.12.014”

The publisher apologizes for these mistakes. The original article has been updated.

